# Short-Duration Swimming Exercise after Myocardial Infarction Attenuates Cardiac Dysfunction and Regulates Mitochondrial Quality Control in Aged Mice

**DOI:** 10.1155/2018/4079041

**Published:** 2018-04-11

**Authors:** Dajun Zhao, Yang Sun, Yanzhen Tan, Zhengbin Zhang, Zuoxu Hou, Chao Gao, Pan Feng, Xing Zhang, Wei Yi, Feng Gao

**Affiliations:** ^1^Department of Cardiovascular Surgery, Xijing Hospital, The Fourth Military Medical University, 127 Changle West Road, Xi'an 710032, China; ^2^Department of Geriatric, Xijing Hospital, The Fourth Military Medical University, 127 Changle West Road, Xi'an 710032, China; ^3^Department of Aerospace Medicine, The Fourth Military Medical University, 169 Changle West Road, Xi'an 710032, China; ^4^Department of Cardiology, Xijing Hospital, The Fourth Military Medical University, 127 Changle West Road, Xi'an 710032, China

## Abstract

**Background:**

Exercise benefits to cardiac rehabilitation (CR) following stable myocardial infarction (MI). The suitable exercise duration for aged patients with coronary heart disease (CHD) remains controversial, and the underlying molecular mechanism is still unclear.

**Methods and Results:**

18-Month-old mice after stable MI were randomly submitted to different durations of exercise, including 15 and 60 min swimming training (ST) once per day, five times a week for 8 weeks. Compared to sedentary mice, 15 min ST, rather than 60 min ST, significantly augmented left ventricular function, increased survival rate, and suppressed myocardial fibrosis and apoptosis. 15 min ST improved mitochondrial morphology via regulating mitochondrial fission-fusion signaling. 15 min ST regulated mitophagy signaling via inhibiting LC3-II and P62 levels and increasing PINK/Parkin expression. 15 min ST also inhibited ROS production and enhanced antioxidant SOD2 activity. Notably, 15 min ST significantly increased sirtuin (SIRT) 3 level (2.7-fold) in vivo while the inhibition of SIRT3 exacerbated senescent H9c2 cellular LDH release and ROS production under hypoxia. In addition, SIRT3 silencing impairs mitochondrial dynamics and mitophagy in senescent cardiomyocytes against simulated ischemia (SI) injury.

**Conclusion:**

Collectively, our study demonstrated for the first time that sustained short-duration exercise, rather than long-duration exercise, attenuates cardiac dysfunction after MI in aged mice. It is likely that the positive regulation induced by a short-duration ST regimen on the elevated SIRT3 protein level improved mitochondrial quality control and decreased apoptosis and fibrosis contributed to the observed more resistant phenotype.

## 1. Introduction

Physical exercise is an effective therapy for patients with stable coronary heart disease (CHD). Exercise-based cardiac rehabilitation (CR) reduces all-cause mortality, declines rehospitalization, and improves health-related quality of life following myocardial infarction (MI) [1–3]. However, aging greatly influences exercise parameters and cardiac performances [4]. Elderly patients with CHD are more prone to deconditioning, frailty, and disability with intrinsic muscle weakness, joint instability, and metabolic risks, leading to the difficult implementation of exercise. Moreover, the association between the duration of exercise and the cardioprotective effects remains controversial [5, 6]. Whether aged heart benefits distinctly from different durations of exercise as rehabilitative modalities is not yet understood.

Among the complex determinants of aging, mitochondrial dysfunction is identified as one of the major causes [7]. The maintenance of an adequate pool of functional mitochondria is crucial for cardiomyocyte homeostasis [8]. Mitochondrial dysfunction triggers mitochondrial quality control to remove damaged components, recover mitochondrial morphology, and repair cardiomyocyte function responding to stresses [9]. Mitochondrial quality control consists of a series of mechanisms, including reactive oxygen species (ROS) scavenging, mitochondrial dynamics, and mitophagy. Studies have revealed that exercise improves mitochondrial quality both in healthy [10] and in metabolic and neurodegenerative disorders as well as in aging [11, 12].

Sirtuin, also known as Sir2 proteins, is a family of nicotinamide adenine dinucleotide-dependent deacetylases. SIRT3 is one of the sirtuin family members that localizes in mitochondria [13]. It inhibits ROS production and maintains mitochondrial functions [14, 15]. Studies have demonstrated that SIRT3 protects against acute myocardial ischemia injury [16] while SIRT3 deficiency significantly inhibits angiogenesis and cardiac functional recovery following MI [17]. Notably, SIRT3 expression is proved to be modified by exercise training both in humans and murines [18], suggesting that SIRT3 might be crucial to exercise's rehabilitative effects against MI injury consequences.

The aims of this study were (1) to determine whether exercise rehabilitates cardiac dysfunction after MI in aged mice; (2) to investigate the suitable exercise duration for CR; (3) to elucidate whether mitochondrial quality control is associated with rehabilitative effects of exercise after MI; and (4) to examine the potential role for SIRT3 in the process.

## 2. Materials and Methods

All animal experiments were approved by the Fourth Military Medical University Committee on Animal Care. C57BL/6J male mice were provided by the Experimental Animal Center of the Fourth Military Medical University (Xi'an, Shaanxi, China). Mice were maintained in 12 h : 12 h artificial light-dark cycles, with lights on at 6 : 00 AM, and were housed individually in the lab's animal room. H9c2 cardiac cell lines were purchased from the American Type Culture Collection (ATCC®CRL-1446™).

### 2.1. Mouse Model of MI

18-Month-old male C57BL/6J mice were anesthetized with 2% isoflurane. Myocardial infarction (MI) was produced via the ligation of left coronary artery as previously described [19]. Sham-operated control mice (21 mice) underwent the same procedures except that the suture placed under the left coronary artery was not tied.

### 2.2. Exercise Protocol

Four months after stable MI, 68 alive mice were randomly divided into three groups: (1) sedentary (MI-sedentary, 22 mice); (2) short-duration swimming training (ST) (MI + 15' ST, 23 mice); and (3) long-duration ST (MI + 60' ST, 23 mice). The ST protocol was adapted from previously published procedures [20, 21]. Briefly, mice in groups of three to five animals were trained 5 days per week for 8 weeks in the 60 cm × 90 cm buckets filled with ≈20 cm depth of water at 33–35°C. The ST lasted 15 min on the first day. Then the exercise duration was maintained at 15 min/day in the MI + 15' ST group, or progressively increased to 60 min/day in the MI + 60' ST group during the first week. All training sessions took place during the morning hours (9 : 00 AM–11 : 00 AM). Mouse physical parameters and the survival rate were assessed before and during the ST period. Meanwhile, sedentary animals were housed individually in cages as a control.

### 2.3. Echocardiography

Mice (8-9 per group) were submitted to transthoracic echocardiography (VisualSonics Vevo 2100 Imaging System) to evaluate the cardiac structure and function before and during the ST period. M-mode tracings were taken and echocardiogram analysis was performed.

### 2.4. Masson Trichrome Staining

Mouse hearts (6–8 mice per group) were perfused with ice-cold phosphate buffered saline (PBS) and were fixed with 4% paraformaldehyde, embedded in paraffin, and coronally sectioned (3–6 *μ*m thick). Sections were stained using a Masson trichrome stain assay (Sigma-Aldrich, HT15-1KT) according to the manufacturer's instructions.

### 2.5. TUNEL Staining

Mouse hearts (6–8 mice per group) were fixed with 4% paraformaldehyde. Sections were stained with TUNEL staining assay (Roche Diagnostics Corporation, 11684817910) and *α*-actin antibody (Sigma-Aldrich, A7811) to detect myocardial apoptotic cells according to the manufacturer's instructions.

### 2.6. Detection of ROS Production

Mouse hearts (6–8 mice per group) were embedded in optimal cutting temperature compound (OCT) and were frozen immediately after euthanasia. Frozen sections (7–10 *μ*m thick) were cut by a cryomicrotome (Model CM3050S, Leica Microsystems) and incubated with dihydroethidium (DHE, 2 mM; Molecular Probes, D-1168) for 1 hour at room temperature. Slides were examined by a laser scanning confocal microscope (FluoView™ FV1000, Olympus). The numbers of DHE-positive nuclei and the total nuclei were counted (100 fields per group).

Cardiomyocyte mitochondrial superoxide generation was assessed using the MitoSOX Red dye (Molecular Probes, Invitrogen, M36008). Living cells were incubated with 5 mM MitoSOX Red at 37°C for 10 min and were examined by the laser scanning confocal microscope.

### 2.7. Transmission Electron Microscopy

Left ventricular tissues (6 mice per group) were fixed with 2.5% glutaraldehyde in 0.1 M sodium phosphate (pH 7.4) overnight at 4°C. After postfixation in 1% OsO_4_, samples were dehydrated through graded alcohols and embedded in Epon Araldite. Ultrathin sections (50 nm) were cut by an ultramicrotome (Ultracut E, Leica) and stained with uranyl acetate and lead citrate. The specimens were viewed on a Tecnai G2 Spirit electron microscope (FEI Co., Hillsboro, OR). Images were captured at 15,000x magnification. Mitochondrial shape descriptors (including aspect ratio, circularity, roundness, and solidity) and area measurements were obtained using ImageJ (version 1.42q, National Institutes of Health, Bethesda, MD). A total of 243 mitochondria from the sham group, 257 mitochondria from the MI-sedentary group, 240 mitochondria from the MI + 15' ST group, and 251 mitochondria from the MI + 60' ST group were analyzed from five electron micrographs. The frequency distribution of the mitochondrial area was determined by GraphPad Prism 6 statistic software, yielding frequency histograms.

### 2.8. Isolation of Mitochondria

Mitochondria were isolated using mitochondria isolation kits (Beyotime Institute of Biotechnology, c3606 for tissue and c3601 for cell) according to the manufacturer's instructions. Briefly, fresh mouse hearts (6 to 8 mice per group) were minced in the centrifuge tube. The pellet was homogenized in the trypsin-EDTA (8 *μ*L/mg) solution and centrifuged at 600*g* for 20 s. After washing, the pellet was resuspended with 8 *μ*L/mg mitochondria separation reagent B combined with PMSF and transferred into a prechilled glass homogenizer. The suspension was homogenized for 20–30 times on the ice and centrifuged at 600*g* for 5 min. Mitochondria were pelleted from the supernatant via centrifugation at 11,000*g* for 10 min. The final pellet was either resuspended in 40 *μ*L/mg mitochondria store liquid for intact function assay or lysed for Western blot analysis.

For cellular mitochondria isolation, H9c2 cardiomyocytes at a density of 5 × 10^7^ cells were harvested and resuspended in a 2.5 mL mitochondria isolation buffer. The suspension was homogenized for 15–20 times in the glass homogenizer and centrifuged at 600*g* for 10 min. Then, the supernatant was centrifuged at 11,000*g* for 10 min. The final mitochondrial pellet was lysed for Western blot analysis.

### 2.9. Induction of Cell Senescence and SA-*β*-gal Staining

H9c2 cardiomyocytes were cultured in high-glucose Dulbecco's modified Eagle's medium (DMEM) supplemented with 10% fetal bovine serum (FBS), 100 U/mL penicillin, and 100 mg/mL streptomycin at 37°C with 5% CO_2_. Doxorubicin (DOX, Sigma-Aldrich, D1515) was diluted with 5% glucose immediately before use. Cultured cells were treated with DOX (0.1 *μ*M) for 24 h to induce senescence and 5% glucose was used as a vehicle control. Senescence-associated-*β*-galactosidase (SA-*β*-gal, Beyotime Institute of Biotechnology, C0602) staining was used to detect cell senescence as previously described [22–24]. The number of SA-*β*-gal-positive cells was determined by a microscope (×100) and expressed as a percentage of all counted cells (Suppl.  Figure 1A and B).

### 2.10. In Vitro Lentivirus-Mediated SIRT3 Silencing

H9c2 cardiomyocytes were grown in a 6-well plate to approximate 80% confluence (≈5 × 10^4^ cells). Cultured cells were transfected with SIRT3-ShRNA lentivirus (5 × 10^8^ Tu/mL, Shanghai GenePharma Co. Ltd., Lv3-SIRT3-Rat-597) or a negative control (NC, 1 × 10^8^ Tu/mL, Shanghai GenePharma Co. Ltd., Lv3NC) at 100 multiply of infection (MOI) in the presence of 5 *μ*g/mL polybrene according to the previously published studies [25, 26]. Cells infected with GFP-SIRT3 lentivirus were observed under a microscope (×100). The silence efficiency was determined by Western blot.

### 2.11. Simulated Ischemia (SI)

Normal culture medium was replaced with Hank's balanced salt solution (HBSS, Gibco, 14025076), and cardiomyocytes were placed in a Napco 8000WJ hypoxia (1% O_2_, 5% CO_2_, and 94% N_2_) incubator (Thermo Scientific) for 12 h as previously described [27].

### 2.12. Cell Viability

H9c2 cellular viability was analyzed after SIRT3 silencing followed by hypoxia. CCK-8 assay (Sigma-Aldrich, 96992) was used according to the manufacturer's instructions. Briefly, cells were incubated with 5 mg/mL CCK-8 in a CO_2_ incubator for 3 h. The medium was aspirated, and the absorbance of each well was measured by the plate reader at a test wavelength of 460 nm with a reference wavelength of 630 nm. Optical density (OD) was utilized as the indicator of the cell survival rate.

### 2.13. Western Blot Analysis

Whole heart lysate and mitochondrial lysate were used in the present study. The protein concentrations were determined by a BCA protein assay kit (Thermo Fisher, 23225). Proteins were separated through electrophoresis and transferred to PVDF membranes. The membranes were blocked for 2 hours in 5% nonfat dry milk and were subsequently incubated overnight at 4°C with appropriate primary antibodies against Drp1 (Cell Signaling Technology, number 8570), Fis1 (Enzo Life Sciences, ALX-210-1037), Mfn1 (Santa Cruz Biotechnology, sc-166644), Mfn2 (Sigma-Aldrich, M9073), Opa1 (BD Biosciences, 612606), COX IV (Cell Signaling Technology, number 4844), LC3A/B (Cell Signaling Technology, number 2775), P62 (Cell Signaling Technology, number 39749), PINK1 (Cell Signaling Technology, number 6946), Parkin (Cell Signaling Technology, number 4211), SIRT3 (Abgent, number AP6242a), GAPDH (CMCTAG Inc., AT0002), HSP60 (Cell Signaling Technology, number 12165), PGC1-*α* (Cell Signaling Technology, number 2178), SOD2 (Abcam, ab13533), and Ac-SOD2 (Abcam, ab137037). The membranes were washed by Tris-buffered saline containing 0.1% Tween 20 (TBST, pH 7.6) and then subsequently probed with appropriate secondary antibodies (Zhongshan Company, ZB-2301, ZB-2305) at room temperature for 90 min. The protein bands were detected using a Bio-Rad imaging system (Hercules, CA, USA) and normalized to COX IV or GAPDH.

### 2.14. Statistical Analysis

All values in the text and figures are presented as the mean ± standard error of the mean (SEM) of *n* independent experiments. The data were analyzed using GraphPad Prism 6 statistic software (La Jolla, CA, USA). Data were submitted to *t*-test (two groups) or one-way ANOVA (three or more groups). Data of cellular experiment were determined with two-way ANOVA followed by post hoc tests with Holm adjustment. *P* values of <0.05 (two sided) were considered to be statistically significant.

## 3. Results

### 3.1. Short-Duration Exercise after MI Attenuates Cardiac Dysfunction and Improves the Survival Rate in Aged Mice

**Figure 1:**
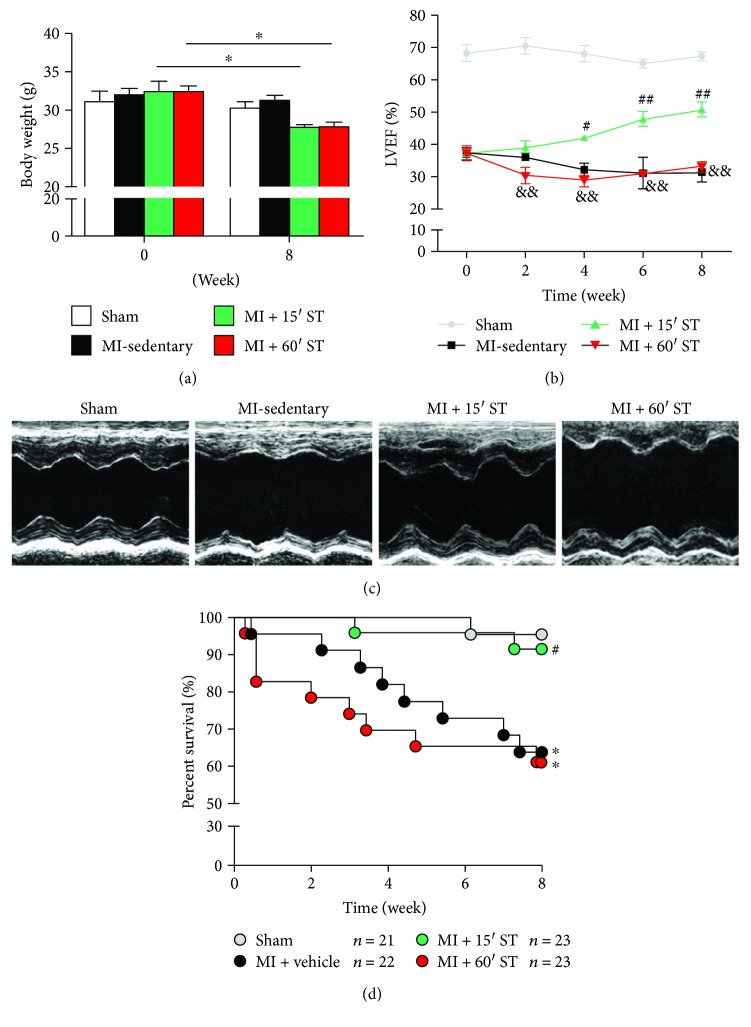
Short-duration exercise after MI promotes left ventricular ejection fraction (LVEF) and reduces mortality in aged mice. (a) Statistics of mouse body weight before and after 8-week swimming training (ST). (b) LVEF analysis. (c) Echocardiography of aged mice after 8-week ST. (d) Survival curves. ^∗^ *P* < 0.05 versus the sham group; ^#^ *P* < 0.05, ^##^ *P* < 0.01 versus the MI-sedentary group; ^&&^ *P* < 0.01 versus the MI + 15' ST group. *N* = 21–23 for survival rate analysis and *N* = 8–9 for other assessments.

To determine exercise's effects upon post-MI injury in aged mice, mouse physical and cardiovascular parameters were measured before and after 8-week ST. Mouse body weight was decreased after swimming for 8 weeks (*P* < 0.05, resp., Figure 1(a)) whereas only 15 min ST mice manifested a lower ratio of heart weight to body weight (*P* < 0.05, Table 1). Echocardiographic results (Figures 1(b) and 1(c)) revealed that post-MI mouse left ventricular ejection fraction (LVEF) was augmented after 15 min ST for 4 weeks (*P* < 0.01) and continued increasing for 8 weeks. Furthermore, 15 min ST improved the mouse post-MI survival rate (*P* < 0.05) compared to the MI-sedentary group (Figure 1(d)). However, 60 min ST mice manifested no significant changes in phenotype. Taken together, these data demonstrate that short-duration rather than long-duration exercise protects against post-MI injury in aged mice.

### 3.2. Short-Duration Exercise after MI Inhibits Cardiac Fibrosis and Apoptosis in Aged Mice

**Figure 2:**
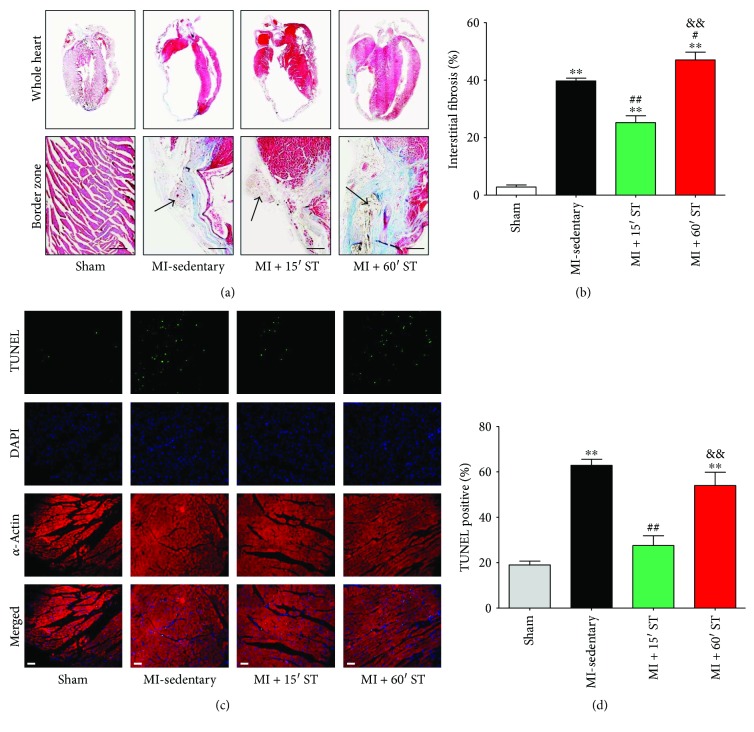
Short-duration exercise after MI inhibits cardiac fibrosis and apoptosis in aged mice. (a, b) Myocardial interstitial fibrosis determined by Masson trichrome staining. Black arrows indicate the ligation of left coronary artery. Scale bars, 50 *μ*m. (c, d) Cardiomyocyte apoptosis determined by TUNEL staining. Scale bars, 30 *μ*m. ^∗∗^ *P* < 0.01 versus the sham group; ^#^ *P* < 0.05, ^##^ *P* < 0.01 versus the MI-sedentary group; ^&&^ *P* < 0.01 versus the MI + 15' ST group. *N* = 6–8.

Then, we investigated whether different durations of ST affected cardiac fibrosis and apoptosis, the primary causes of cardiac remodeling and heart failure. 15 min ST mice manifested a lower myocardial interstitial fibrosis (*P* < 0.01, Figures 2(a) and 2(b)) and less myocardial apoptosis (*P* < 0.01, Figures 2(c) and 2(d)) compared to the MI-sedentary group. However, 60 min ST mice performed an increase of myocardial interstitial fibrosis (*P* < 0.05, Figures 2(a) and 2(b)) with no change of cardiac apoptosis (*P* = 0.16, Figures 2(c) and 2(d)). In summary, short-duration exercise attenuates postischemic cardiac fibrosis and apoptosis in aged mice.

### 3.3. Short-Duration Exercise after MI Restores Mitochondrial Morphology and Regulates Mitochondrial Dynamics Signaling in Aged Heart

**Figure 3:**
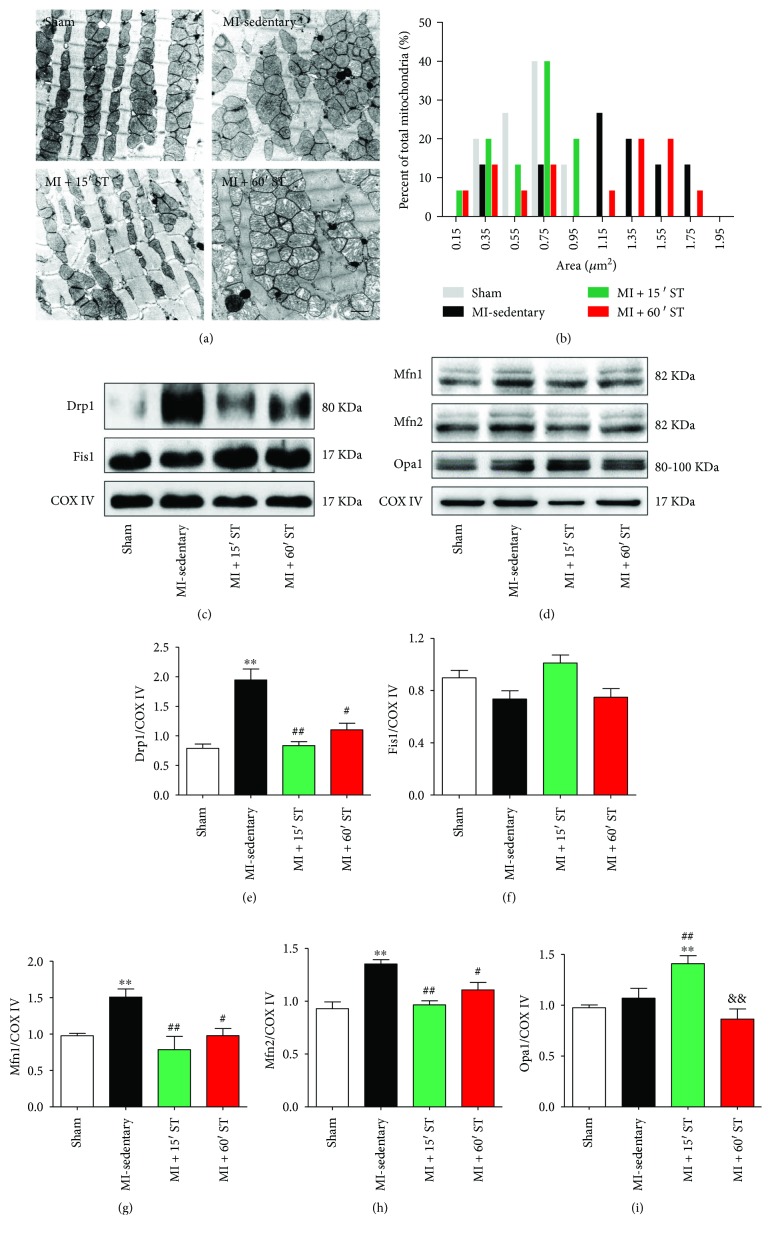
Short-duration exercise after MI modulates mitochondrial morphology and dynamics in aged heart. (a) Representative transmission electron micrographs of cardiac mitochondria. Scale bars, 1 *μ*m. (b) Frequency distribution (% total mitochondria) of the mitochondrial surface area. *N* = 243–251. (c–i) Western blot analysis of mitochondrial fission and fusion markers. ^∗∗^ *P* < 0.01 versus the sham group; ^#^ *P* < 0.05, ^##^ *P* < 0.01 versus the MI-sedentary group; ^&&^ *P* < 0.01 versus the MI + 15' ST group. *N* = 6–8.

Mitochondrial impairment directly contributes to aging and age-related diseases; we examined cardiac mitochondrial ultrastructure by transmission electron microscopy (TEM). As shown in Figure 3(a), mitochondria in the MI-sedentary group were disorganized and in large clusters with small, round mitochondria. Mitochondrial shape was distinctly alerted with the declined aspect ratio (*P* < 0.01) and increased circularity (*P* < 0.05) and roundness (*P* < 0.01) compared to that in the sham group (Table 2). Moreover, the frequency distribution for mitochondrial size was highly skewed post-MI (Figure 3(b)). 15 min ST improved mitochondria to be more homogeneous in size (Figures 3(a) and 3(b)).

However, a large number of swelling mitochondria with broken or dismissing ridge were observed in 60 min ST mouse hearts (Figure 3(a)).

To determine whether mitochondrial dynamics contribute to the impaired mitochondrial morphology, we quantified mitochondrial fission-fusion signaling markers. Aged mice after MI manifested significant increases of fission-related protein Drp1 (2.5-fold) and fusion-related protein Mfn1 and Mfn2 levels (*P* < 0.01, resp., Figures 3(c)–3(e), 3(g) and 3(h)), in consistent with the TEM results. 15 min ST reduced the levels of Mfn1, Mfn2, and Drp1 (*P* < 0.01, resp.) while increased Opa1 expression (*P* < 0.01). Differently, 60 min ST inhibited Mfn1, Mfn2, and Drp1 expression (*P* < 0.05, resp.) with no alteration of Opa1 (Figures 3(c)–3(e), 3(g)–3(i)). However, no changes of fission-related Fis1 levels (Figures 3(c) and 3(f)) were detected in each group. These results suggest that short-duration exercise after MI regulates mitochondrial fission-fusion signaling to recover mitochondrial morphology in aged mice.

### 3.4. Short-Duration Exercise after MI Regulates Mitophagy Signaling in Aged Mice

**Figure 4:**
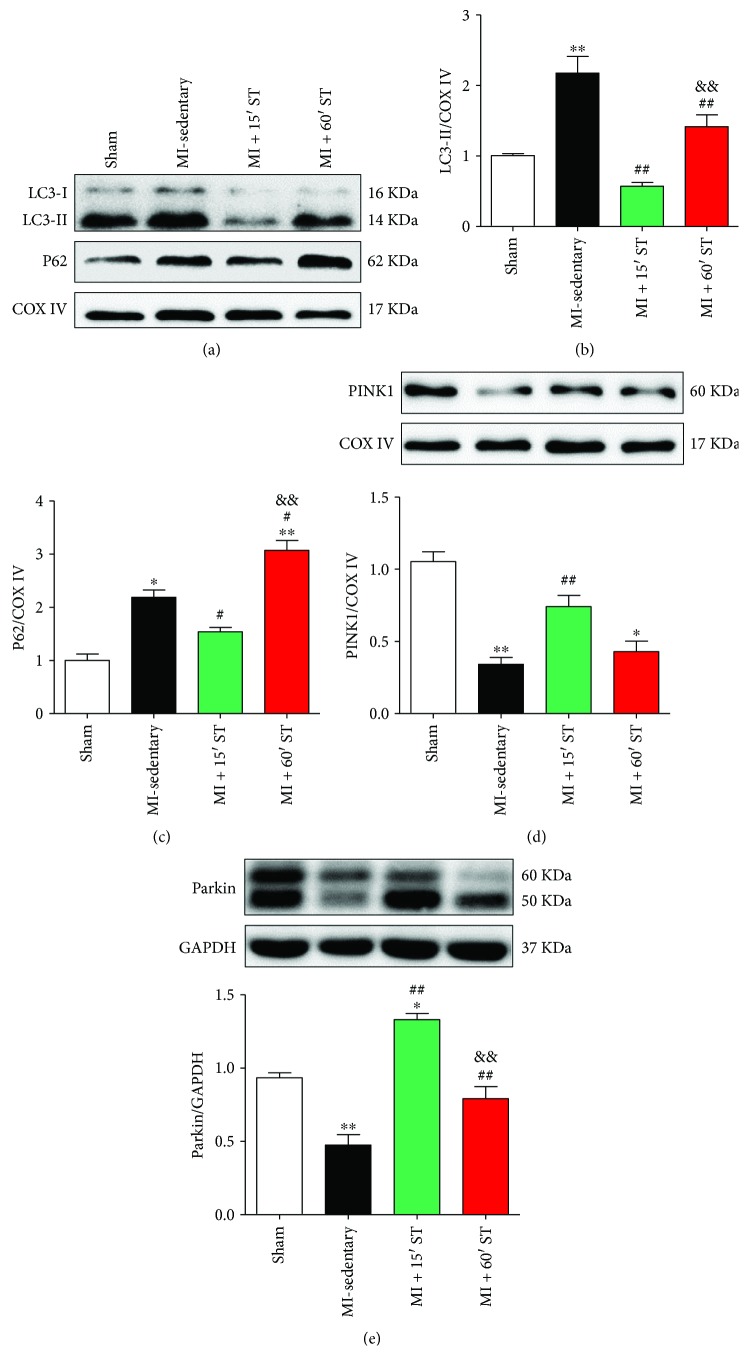
Short-duration exercise after MI regulates mitophagy signaling in aged mice. (a–c) Western blot analysis of mitophagy markers LC3 and P62. (d-e) Western blot analysis of PINK1 and Parkin. ^∗^ *P* < 0.05, ^∗∗^ *P* < 0.01 versus the sham group; ^#^ *P* < 0.05, ^##^ *P* < 0.01 versus the MI-sedentary group; ^&&^ *P* < 0.01 versus the MI + 15' ST group. *N* = 6–8.

To evaluate exercise's effect on mitophagy, we quantified the expression of mitophagy signaling-related markers. The levels of mitochondrial LC3-II were increased in MI-sedentary mice (*P* < 0.01) and 60 min ST mice (*P* < 0.01) but were declined in 15 min ST mice (*P* < 0.01) compared to that in the sham group (Figures 4(a) and 4(b)). Levels of p62 manifested similar changes on ST (Figures 4(a) and 4(c)). The increases of LC3-II levels might derive from excessive mitophagy or from downstream block of autophagic vacuole processing. To distinguish between these 2 possibilities, we assessed PINK1 and Parkin expression, two proteins involved in mitophagy initiation. We found that MI injury suppressed PINK1 and Parkin expression (*P* < 0.01, resp.). Their levels were restored when exposed to 15 min ST (*P* < 0.01, resp.) rather than 60 min ST (Figures 4(d) and 4(e)). These data suggest that short-duration exercise after MI regulates mitophagy signaling biomarkers in aged heart.

### 3.5. Short-Duration Exercise after MI Attenuates Oxidative Stress and Increases Mitochondrial SIRT3 Expression in Aged Heart

**Figure 5:**
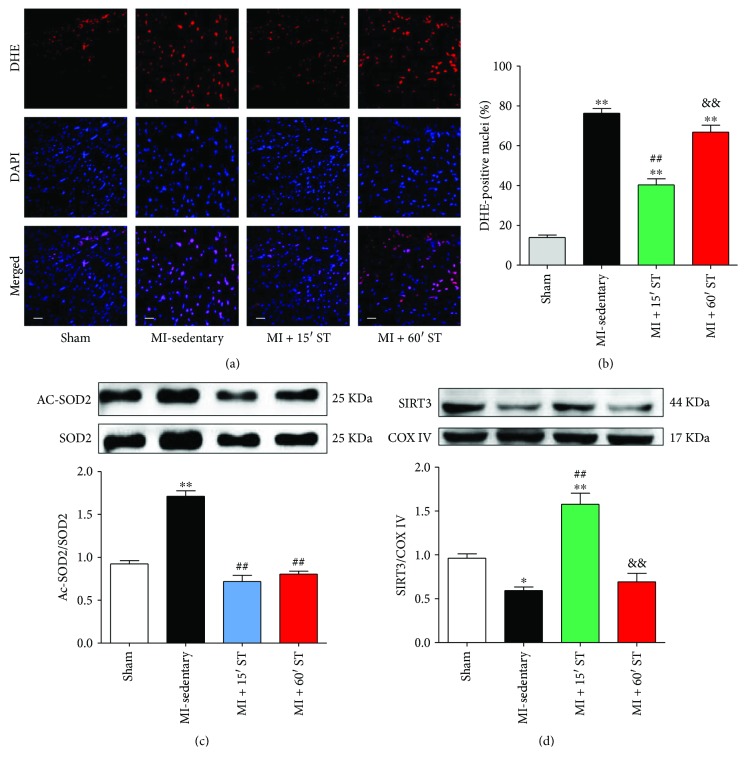
Short-duration exercise after MI attenuates oxidative stress and increases SIRT3 expression in aged heart. (a) ROS production determined by DHE staining. Scale bars, 50 *μ*m. (b) Statistical analysis of DHE positive cells. (c) Ac-SOD2 to SOD2 ratio. (d) Western blot analysis of mitochondrial SIRT3. ^∗^ *P* < 0.05; ^∗∗^ *P* < 0.01 versus the sham group; ^##^ *P* < 0.01 versus the MI-sedentary group; ^&&^ *P* < 0.01 versus the MI + 15' ST group. *N* = 6–8.

Furthermore, we tested exercise's effects on oxidative stress. Aged mice after MI manifested a great accumulation of superoxide compared to the sham group (4.6-fold, *P* < 0.01). 15 min ST distinctly inhibited ROS production for 48.3% (*P* < 0.01) while 60 min ST slightly reduced 15.9% ROS production (*P* < 0.01) (Figures 5(a) and 5(b)). Moreover, 15 min ST mice manifested higher antioxidant SOD2 activity determined by the decrease of SOD2 acetylation compared to the MI-sedentary group (*P* < 0.01, Figure 5(c)). Together, these findings support that short-duration exercise protects against post-MI injury via antioxidant actions in aged mice. Notably, mitochondrial SIRT3 expression was increased 2.7-fold under 15 min ST (*P* < 0.01), but with no change via 60 min ST (*P* = 0.81), compared to that in the MI-sedentary group (Figure 5(d)). It indicates that SIRT3 might play a critical role in short-duration exercise's cardioprotections.

### 3.6. SIRT3 Deficiency Exacerbates SI-Induced Senescenced Cardiomyocyte Apoptosis and ROS Production In Vitro

**Figure 6:**
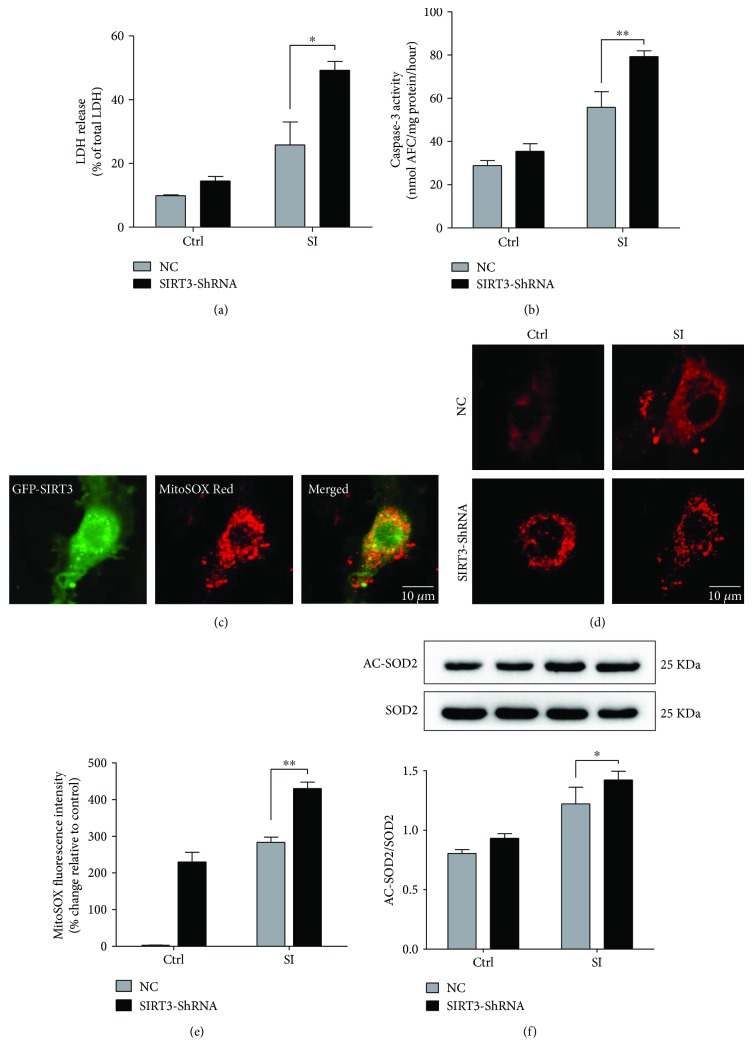
SIRT3 deficiency exacerbates SI-induced cardiomyocyte apoptosis and oxidative stress. (a) Effect of SIRT3 silencing on cell death, determined by LDH release assay. (b) Effect of SIRT3 silencing on apoptotic events, determined by caspase-3 activity assay. (c–e) Effect of SIRT3 silencing on mitochondrial ROS production, determined by MitoSOX Red dye. Scale bars, 10 *μ*m. (f) Ac-SOD2 to SOD2 ratio upon SIRT3 inhibition. ^∗^ *P* < 0.05, ^∗∗^ *P* < 0.01 versus the SI + NC group. *N* = 3–6.

To further determine the cardioprotective role of SIRT3 in aging, we used lentivirus to knockdown SIRT3 expression in H9c2 cardiomyocytes. Compared to negative control (NC), the level of cellular SIRT3 was dramatically decreased by 74.5% in the SIRT3-ShRNA group (*P* < 0.01, Suppl.  Figure 1C and D), confirming the lentivirus silence efficiency. Cell viability was measured via CCK-8 assay, and the inhibition of SIRT3 expression significantly increased SI-induced cell death (*P* < 0.01, Suppl.  Figure 1E). To confirm our finding, LDH release and caspase-3 activity were performed. In the SI + NC group, cellular LDH release was approximately 25.8% under hypoxia while in the SIRT3-ShRNA group, it significantly raised to 49.2% (*P* < 0.05, Figure 6(a)). Caspase-3 activity manifested similar changes under SIRT3 deficiency (*P* < 0.01, Figure 6(b)). Moreover, mitochondrial ROS production was increased in senescenced cardiomyocytes exposed to hypoxia. The inhibition of SIRT3 exacerbated superoxide generation compared to the SI + NC group (*P* < 0.01, Figures 6(c)–6(e)). The SOD2 activity was impaired by hypoxia and was even low with SIRT3 inhibition (*P* < 0.05, Figure 6(f)). These results demonstrate that SIRT3 protects against SI injury in senescenced cardiomyocytes via antiapoptotic and antioxidative actions.

### 3.7. SIRT3 Regulates Mitochondrial Dynamics and Mitophagy Signaling under SI Injury

**Figure 7:**
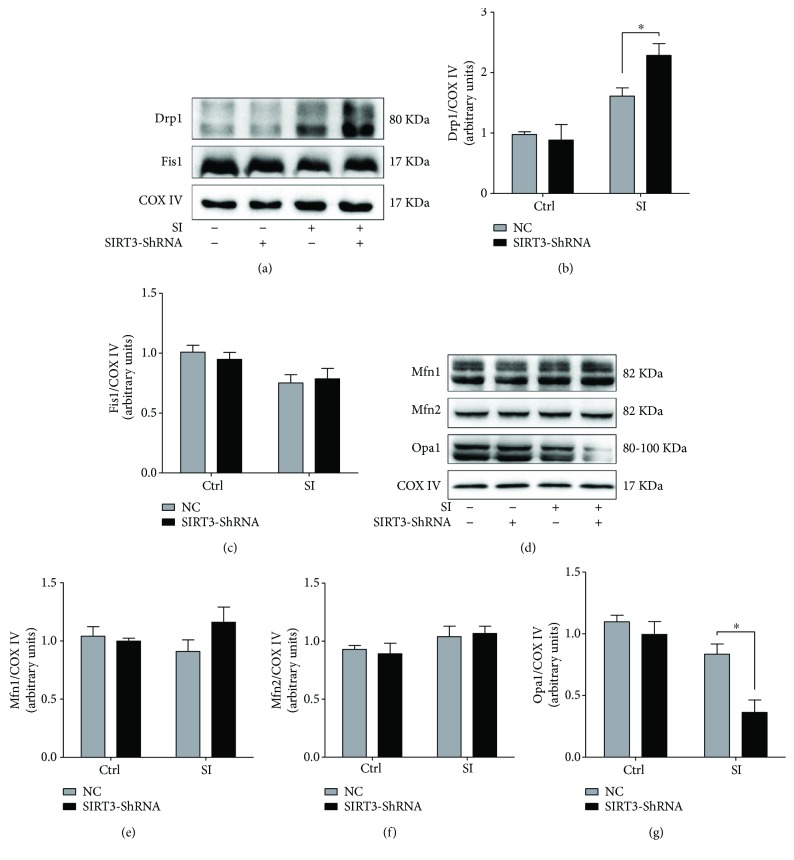
SIRT3 regulates cardiomyocytes mitochondrial dynamics under SI injury. (a–c) Western blot analysis of fission proteins Drp1 and Fis1 upon SIRT3 silencing. (d–g) Western blot analysis of fusion proteins Mfn1, Mfn2, and Opa1 upon SIRT3 silencing. ^∗^ *P* < 0.05 versus the SI + NC group. *N* = 3–6.

**Figure 8:**
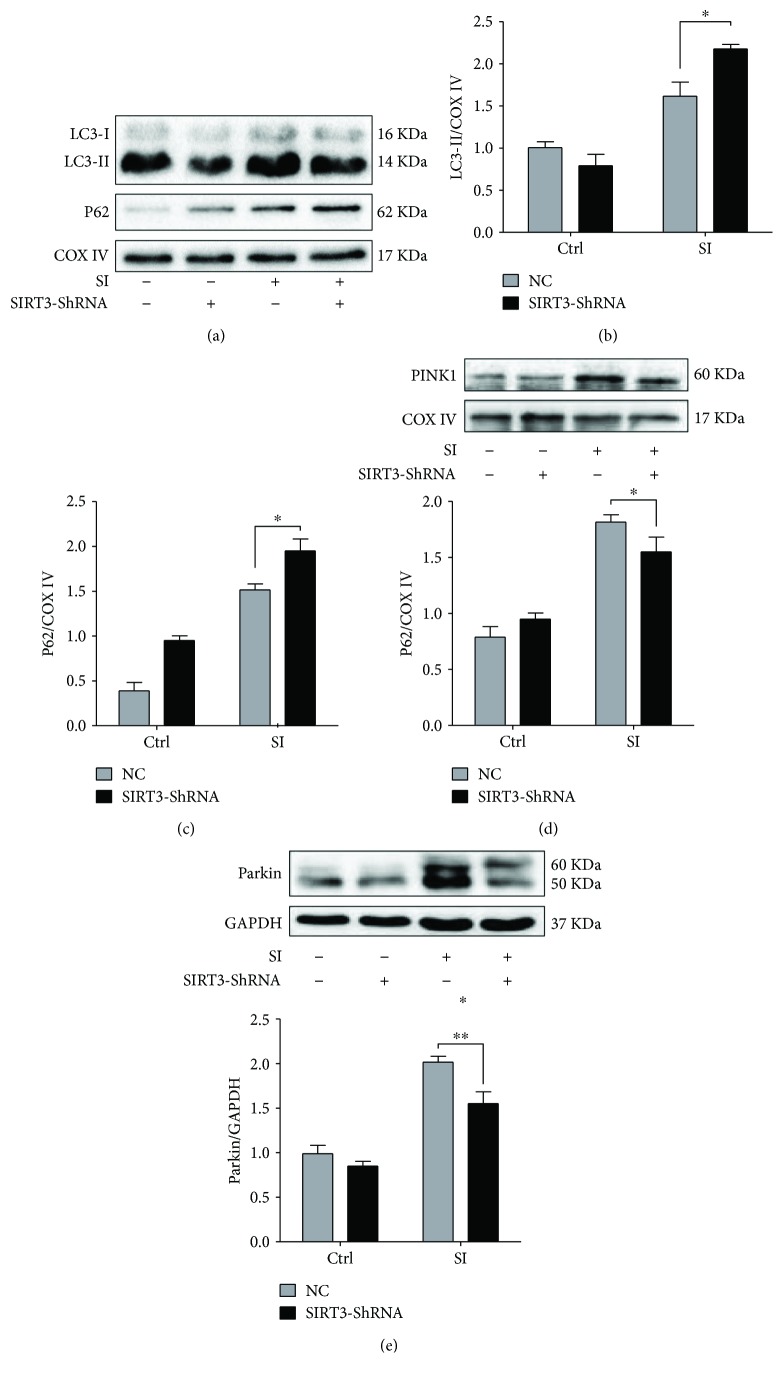
SIRT3 regulates SI-induced mitophagy signaling in vitro. (a–c) Western blot analysis of mitophagy makers LC3 and P62 upon SIRT3 silencing. (d-e) Western blot analysis of PINK1 and Parkin upon SIRT3 silencing. ^∗^ *P* < 0.05, ^∗∗^ *P* < 0.01 versus the SI + NC group. *N* = 3–6.

Finally, we measured mitochondrial dynamics and mitophagy signaling markers upon SIRT3 silencing. Compared to the SI + NC group, the level of Drp1 was significantly raised to 41.6% (*P* < 0.05, Figures 7(a) and 7(b)) and Opa1 expression was reduced (*P* < 0.05, Figures 7(d) and 7(g)) under SIRT3 inhibition following hypoxia. However, no obvious changes were observed on Mfn1, Mfn2, and Fis1 (Figures 7(a) and 7(c)–7(f)) expression upon SIRT3 silencing. SIRT3 deficiency manifested the upregulation of LC3-II (*P* < 0.05) and P62 levels (*P* < 0.05) compared to the SI + NC group (Figures 8(a)–8(c)). Meanwhile, SIRT3 deficiency suppressed PINK1 and Parkin expression under hypoxia (*P* < 0.05, *P* < 0.01, resp., Figures 8(d) and 8(e)), demonstrating that the inhibition of SIRT3 blunted mitophagy progress under hypoxia.

## 4. Discussion

Cardiac remodeling following MI is associated with left ventricular dysfunction, cardiac hypertrophy, myocardial interstitial fibrosis, and cardiomyocyte apoptosis, which finally lead to decompensated heart failure [28]. Exercise-based cardiac rehabilitation is an effective therapy in attenuating post-MI remodeling [2, 29]. However, some studies exhibited that training with a high volume (duration) led to a lower all-cause mortality [5, 30] while others displayed excessive exercise significantly increased cardiovascular mortality [6]. The proper exercise duration, especially for aged patients with CHD, remains as a question of debate [31, 32]. Furthermore, the underlying molecular mechanism is still unclear.

ST presents a better redistribution of blood flow without significant variations in the cardiac output and heart rate than treadmill or voluntary wheel running programs [33]. Moreover, swimming is much easier for the elderly joints due to reduced effects of gravity. In this study, we assessed two different durations (75 min/week and 300 min/week) of ST's rehabilitative effects on aged mice following stable MI and demonstrated for the first time that short-duration rather than long-duration exercise after MI attenuated cardiac dysfunction and improved the survival rate in aged mice via inhibition of myocardial fibrosis and apoptosis. We suggest that the discrepancies result on exercise duration from several factors. First, clinical studies using a questionnaire have a certain degree of limitation, including undetailed exercise interventions and the subjectivity of the reports. Patients' baseline differences and societal factors also vary from different studies [5]. Moreover, advanced age contributes to a general trend towards the decrease in exercise tolerance [34].

Mitochondria are deeply involved in the aging process [35]. Previous studies suggest a causative link between mitochondrial dysfunction and major phenotypes associated with aging [36, 37]. Aged mitochondria are more susceptible to ischemia injury while regular exercise is demonstrated to improve mitochondrial quality and quantity [10, 38]. In the current study, we observed impaired mitochondrial morphology in aged mice post-MI. A significant increase of swollen mitochondria contributes to the larger mitochondrial surface area while MI-induced excessive mitochondrial fission may lead to the increase of small, round mitochondria. Our data revealed that short-duration exercise improved mitochondrial network remodeling via regulation of mitochondrial dynamics signaling-related proteins Drp1 and Opa1. Mitochondrial fragmentation results in loss of ATP synthesis, increased ROS production, and release of proapoptotic proteins, including cytochrome c and BAX [39]. Exercise-stimulated inhibition of Drp1 restores mitochondrial morphology and promotes cardiac function after MI [40, 41] probably due to increased mPTP resistance [42]. Meanwhile, enhancement of Opa1 maintains morphology of cristae and regulates mitochondrial metabolism since Opa1 protects against apoptotic signaling via preventing cytochrome c release independently from mitochondrial fusion [43, 44]. Studies demonstrated that the mutation of Opa1 is associated with reduced oxidative phosphorylation and ATP synthesis in skeletal muscle and human fibroblasts [45, 46].

Next, we found that aged hearts after MI perform an upregulation of mitophagy signaling-related protein LC3-II and P62 levels together with the inhibition of PINK1/Parkin expression. PINK1 is stabilized and recruits Parkin to mitochondria, initiating mitophagy [47]. The low expression of PINK1/Parkin suggests that the accumulation of LC3-II and P62 protein levels is from inhibition of LC3-II turnover, implicating a block either in autophasosome/lysosome assembling or autolysosome degradation. Our data revealed that short-duration exercise regulates mitophagy signaling biomarkers, including activating PINK1/Parkin expression together with decreasing LC3-II and P62 levels.

Moreover, we demonstrated that aged hearts after MI perform increased oxidative stress with accumulated ROS production and reduced antioxidant enzyme SOD2 activity, consistent with another study [48]. MI-induced ROS accumulation may further contribute to our findings of the increased mitochondrial fragmentation and impaired mitochondrial quality control. Our data revealed that short-duration exercise reduces ROS production and enhances the activity of antioxidant enzyme SOD2 by inhibiting its acetylation in aged mice after MI. The inhibition of oxidative stress directly attenuates cardiac fibrosis and apoptosis to protect against post-MI injury.

Finally, we proved that short-duration exercise after MI upregulates mitochondrial SIRT3 expression in aged mice while SIRT3 deficiency exacerbates cardiomyocyte oxidative stress and apoptosis and blocks mitochondrial dynamics and mitophagy signaling under hypoxia in vitro. SIRT3 regulates mitochondrial biogenesis and function and promotes mitochondrial oxidative stress resistance via altering the acetylation level of MnSOD and enhancing its ability to scavenge ROS [49]. In particular, decreased SIRT3 contributes to the susceptibility of aged hearts to myocardial ischemia/reperfusion (MI/R) injury [50]. Previous studies showed that exercise training increases SIRT3 expression in skeletal muscle [18]. However, Karvinen et al. revealed that high capacity running rats had a higher SIRT3 protein content in skeletal muscle compared to low capacity running rats, contributing to lower mitochondrial protein acetylation [51]. The discrepancies between our finding and Karvinen et al.'s finding may attribute to different exercise models.

In conclusion, different durations of exercise may stimulate different mechanical effects. Exercise duration has independent effects on the hormonal response that significantly affects systemic metabolism [52]. Short-duration exercise elicits a great reduction of serum insulin concentrations and insulin resistance [53] while long-duration exercise increases adrenocorticotropic hormone (ACTH), cortisol, and growth hormone [54]. Studies found that long-duration exercise produces a prolonged postexercise oxygen consumption (EPOC), elevating the energy cost [55]. Our study demonstrated for the first time that sustained short-duration ST after MI attenuates cardiac dysfunction in aged mice. It is likely that the positive regulation induced by a short-duration ST regimen on the elevated SIRT3 protein level improved mitochondrial quality control and decreased apoptosis and fibrosis contributed to the observed more resistant phenotype. However, long-duration ST elicits a deleterious response on CR that may attribute to the declined exercise tolerance in aged mice.

## 5. Limitation

Considering exercising condition is hard to mimic in vitro, we only examined the role of SIRT3 on the regulation of mitochondrial quality control and apoptosis in senescent H9c2 cells under hypoxia. SIRT3-knockout mice may be used in further studies to confirm the relationship between SIRT3 and exercise's cardioprotective effects.

## Figures and Tables

**Table 1:** Physical and echocardiographic parameters of aged mice after 8 weeks of swimming training.

Parameter	Mean ± SEM			
	Sham (*n* = 8)	MI-sedentary (*n* = 9)	MI + 15' ST (*n* = 9)	MI + 60' ST (*n* = 8)
*Physical characteristic*				
BW (g)	30.3 ± 0.73	31.2 ± 0.71	27.7 ± 0.35^∗^	27.7 ± 0.66^∗^
Heart (mg)	140 ± 4.7	200 ± 4.7^∗^	130 ± 3.7^##^	180 ± 7.8^∗^^,&&^
HW/BW (mg/g)	4.6 ± 0.22	6.4 ± 0.26^∗^	4.7 ± 0.13^#^	6.6 ± 0.37^∗^^,&^
Tibial length (cm)	2.3 ± 0.04	2.3 ± 0.04	2.3 ± 0.04	2.3 ± 0.03
HW/TL (mg/cm)	61.0 ± 2.44	87.5 ± 3.24^∗^	57.5 ± 1.83^#^	80.0 ± 3.40^∗^^,&^
*Echocardiography*				
LVEF (%)	67.8 ± 2.21	31.3 ± 2.24^∗^	49.5 ± 1.82^∗^^,##^	34.6 ± 2.14^∗^^,&&^
LVFS (%)	38.3 ± 0.98	17.5 ± 1.46^∗^	25.1 ± 1.59^∗^^,#^	19.7 ± 1.47^∗^^,&^
LVEDD (mm)	4.0 ± 0.13	5.2 ± 0.23^∗^	4.4 ± 0.09^#^	5.5 ± 0.30^∗^^,&^
LVESD (mm)	2.3 ± 0.18	3.9 ± 0.16^∗^	2.9 ± 0.09^#^	3.8 ± 0.28^∗^^,&^

^∗^ *P* < 0.05 versus the sham group; ^#^ *P* < 0.05, ^##^ *P* < 0.01 versus the MI-sedentary group; ^&^ *P* < 0.05, ^&&^ *P* < 0.01 versus the MI + 15' ST group. *N* = 8–9. BW, body weight; HW, heart weight; TL, tibial length; LVEF, left ventricular ejection fraction; LVFS, left ventricular fractional shortening; LVEDD, left ventricular end-diastolic diameter; LVESD, left ventricular end-systolic diameter.

**Table 2:** Mitochondrial morphological parameters of aged hearts after 8 weeks of swimming training.

Parameter	Mean ± SEM			
	Sham (*n* = 243)	MI-sedentary (*n* = 257)	MI + 15' ST (*n* = 240)	MI + 60' ST (*n* = 251)
Area (*μ*m^2^)	0.65 ± 0.05	1.17 ± 0.12^∗^	0.64 ± 0.06^#^	1.11 ± 0.15^∗^^,&^
Aspect ratio	1.97 ± 0.11	1.15 ± 0.21^∗∗^	2.35 ± 0.26^∗^^,##^	1.53 ± 0.08^∗^^,&^
Circularity	0.73 ± 0.03	0.88 ± 0.03^∗^	0.67 ± 0.04	0.77 ± 0.02
Roundness	0.52 ± 0.04	0.73 ± 0.05^∗∗^	0.50 ± 0.05^#^	0.68 ± 0.04^&^
Solidity	0.97 ± 0.01	0.84 ± 0.02	0.91 ± 0.01	0.96 ± 0.01

^∗^ *P* < 0.05, ^∗∗^ *P* < 0.01 versus the sham group; ^#^ *P* < 0.05, ^##^ *P* < 0.01 versus the MI-sedentary group; ^&^ *P* < 0.05 versus the MI + 15' ST group. *N* = 243–257.

## Supplementary Materials

Supplementary 1 H9c2 cardiomyocyte senescence and lentivirus-mediated SIRT3 silencing in vitro. (A) Representative images (magnification, ×100) and quantitation of senescence-associated-*β*-galactosidase (SA-*β*-gal) activity in cardiomyocytes treated with doxorubicin (DOX, 0.1 *μ*M). Scale bars, 50 *μ*m. (B) Representative images showing the morphological changes and SA-*β*-gal activity in cardiomyocytes treated with 0.1 *μ*M DOX. Cells were counterstained with Giemsa solution (magnification, ×400). Scale bars, 10 *μ*m. (C) Representative images showing the infection of GFP-lentivirus-SIRT3-ShRNA. Scale bars, 100 *μ*m. (D) Efficiency of SIRT3 silencing in vitro determined by representative Western blot. (E) Cell viability determined by CCK-8 assay. ^∗∗^ *P* < 0.01 versus the NC group. *N* = 3–6.

Supplementary 2 Exercise's effects on mitochondrial biogenesis and matrix markers. (A–C) Western blot analysis of PGC1-*α* and HSP60. ^∗∗^ *P* < 0.01 versus the sham group; ^##^ *P* < 0.01 versus the MI-sedentary group. *N* = 6.
