# Supplementary material for: Short-Duration Swimming Exercise after Myocardial Infarction Attenuates Cardiac Dysfunction and Regulates Mitochondrial Quality Control in Aged Mice
Source: Oxid Med Cell Longev. 2018 Apr 11;2018:4079041. doi: 10.1155/2018/4079041 (PMC5925211; doi:10.1155/2018/4079041)
Supplement: Supplementary 1 — H9c2 cardiomyocyte senescence and lentivirus-mediated SIRT3 silencing in vitro. (A) Representative images (magnification, ×100) and quantitation of senescence-associated-β-galactosidase (SA-β-gal) activity in cardiomyocytes treated with doxorubicin (DOX, 0.1 μM). Scale bars, 50 μm. (B) Representative images showing the morphological changes and SA-β-gal activity in cardiomyocytes treated with 0.1 μM DOX. Cells were counterstained with Giemsa solution (magnification, ×400). Scale bars, 10 μm. (C) Representative images showing the infection of GFP-lentivirus-SIRT3-ShRNA. Scale bars, 100 μm. (D) Efficiency of SIRT3 silencing in vitro determined by representative Western blot. (E) Cell viability determined by CCK-8 assay. ∗∗ P < 0.01 versus the NC group. N = 3–6. [file 4079041.f1.pptx]

## Slide 1
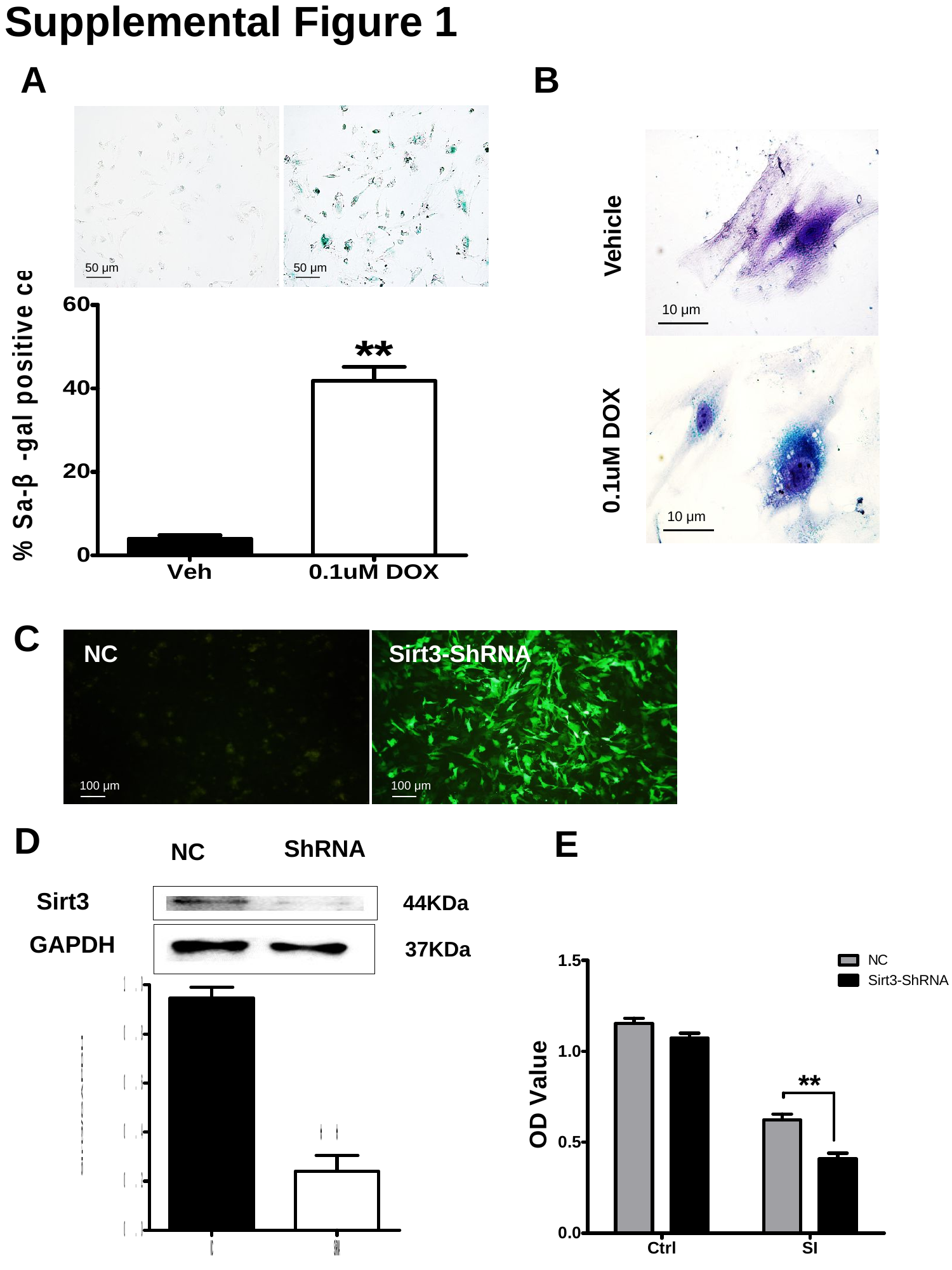

Supplemental Figure 1
A
B
50 μm
50 μm
10 μm
Vehicle
10 μm
0.1uM DOX
C
NC
Sirt3-ShRNA
100 μm
100 μm
D
E
ShRNA
NC
Sirt3
GAPDH
44KDa
37KDa
