# Supplementary figures and images for: Short-Duration Swimming Exercise after Myocardial Infarction Attenuates Cardiac Dysfunction and Regulates Mitochondrial Quality Control in Aged Mice
Source: Oxid Med Cell Longev. 2018 Apr 11;2018:4079041. doi: 10.1155/2018/4079041 (PMC5925211; doi:10.1155/2018/4079041)

## Slide 1
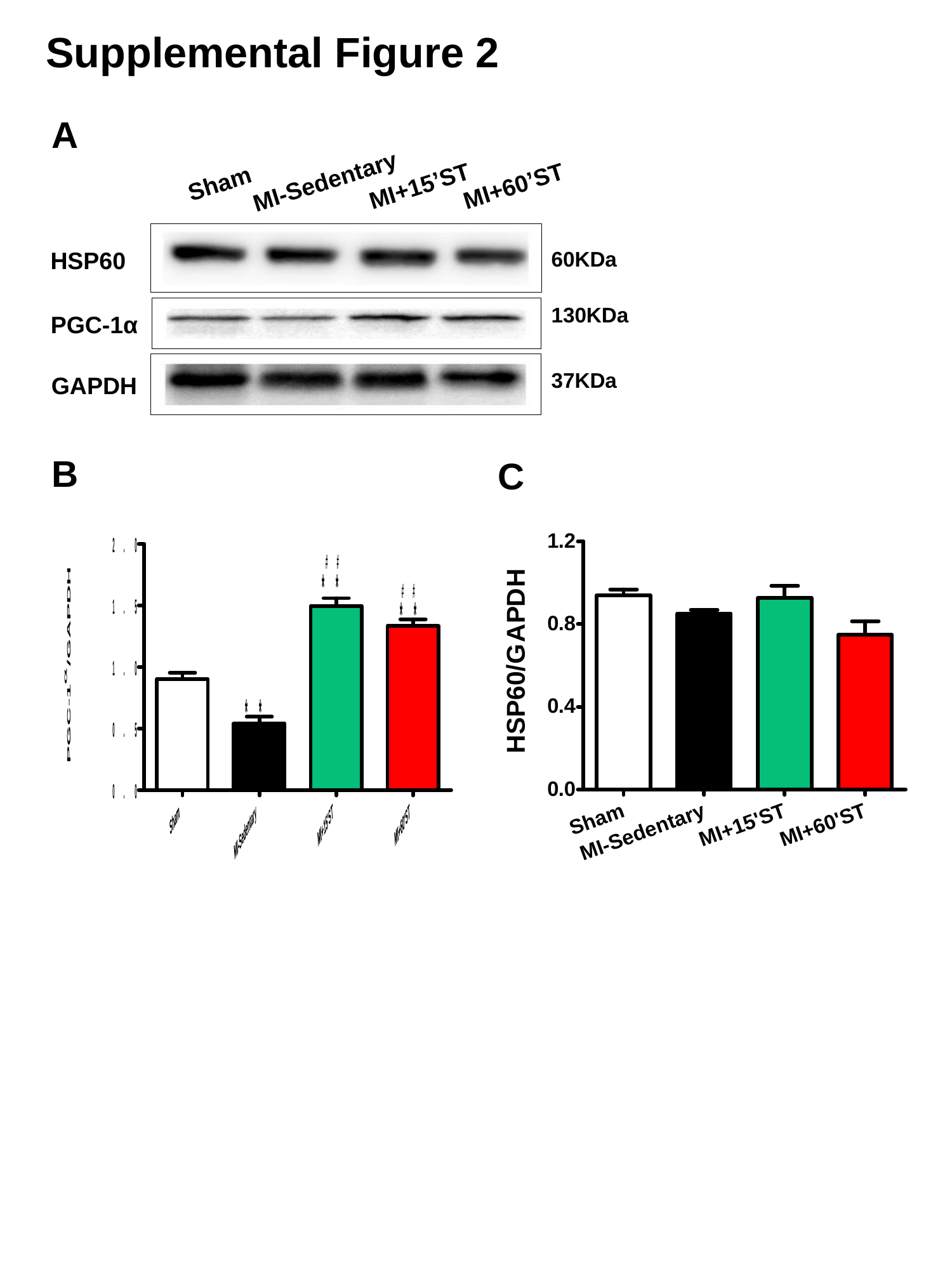

Supplemental Figure 2
A
MI-Sedentary
Sham
MI+15’ST
MI+60’ST
HSP60
60KDa
130KDa
PGC-1α
37KDa
GAPDH
B
C

Supplement: Supplementary 2 — Exercise's effects on mitochondrial biogenesis and matrix markers. (A–C) Western blot analysis of PGC1-α and HSP60. ∗∗ P < 0.01 versus the sham group; ## P < 0.01 versus the MI-sedentary group. N = 6. [file 4079041.f2.pptx]
